# Factors Associated With Mortality in Elderly Hospitalized Patients at Admission

**DOI:** 10.7759/cureus.22709

**Published:** 2022-02-28

**Authors:** Ioannis Vrettos, Panagiota Voukelatou, Stefani Panayiotou, Andreas Kyvetos, Alexandra Tsigkri, Konstantinos Makrilakis, Petros P Sfikakis, Dimitris Niakas

**Affiliations:** 1 Second Department of Internal Medicine, General and Oncology Hospital of Kifissia “Agioi Anargyroi”, Athens, GRC; 2 Internal Medicine, National and Kapodistrian University of Athens School of Medicine, Athens, GRC; 3 First Department of Propaedeutic and Internal Medicine, Laikon General Hospital, Athens, GRC; 4 Health Economics, School of Health Sciences, National and Kapodistrian University of Athens, Athens, GRC

**Keywords:** hospital admission, mortality, elderly persons, qsofa, clinical frailty scale

## Abstract

Background

Several factors have been associated with mortality prediction among older inpatients. The objective of this study was to assess the factors associated with mortality in hospitalized elderly patients.

Methods

A total of 353 consecutively admitted elderly patients (47.9% women), with a median age of 83 years (interquartile range 75.00-88.00), were enrolled in the study and patient characteristics were recorded. Comorbidities were assessed using Charlson Comorbidity Index (CCI), activities of daily living by Barthel Index (BI), frailty was assessed using the Clinical Frailty Scale (CFS), cognition by Global Deterioration Scale (GDS) and symptom severity at admission by quick Sequential Organ Function Assessment (qSOFA) score. CFS, GDS and BI were estimated for the premorbid patients’ status. Parametric and non-parametric tests and binary logistic regression analysis were applied to identify the factors associated with mortality. A receiver operating characteristic (ROC) curve was used to analyse the prognostic value of CFS and qSOFA.

Results

In total, 55 patients (15.6%) died during hospitalization. In regression analysis, the factors associated with mortality were the qSOFA score at admission (p=0.001, odds ratio [OR]=1.895, 95% confidence interval [CI] 1.282-2.802) and the premorbid CFS score (p=0.001, OR=1.549, 95% CI 1.1204-1.994). The classifiers both have almost similar area under the curve (AUC) scores, with CFS performing slightly better. More specifically, both CFS (AUC 0.79, 95% CI 0.73-0.85, p=0.001) and qSOFA (AUC 0.75, 95% CI 0.67-0.83, p<0.001) showed almost the same accuracy for predicting inpatients’ mortality.

Conclusion

This study strengthens the perception of premorbid frailty and disease severity at admission as factors closely related to mortality in hospitalized elderly patients. Simple measures such as CFS and qSOFA score may help identify, in the emergency department, elderly patients at risk, in order to provide timely interventions.

## Introduction

Compared with younger patients, older persons who attend the emergency department are often sicker, more likely to stay longer in the emergency room and more likely to be admitted to the hospital [1]. Moreover, during hospitalization, the mortality rate in elderly patients has been reported to be 4.7-fold higher than in the younger patients [2]. The evaluation of elderlies at the emergency department is complicated because along with the acute pathological conditions that lead them to the hospital, there is also an underlying premorbid health status that plays a significant role [3]. In this time-pressure setting, the early identification of older patients at higher risk of poor outcomes is critical [4]. Identifying those patients may help provide timely interventions to reduce mortality [5].

In previous studies, several factors have been associated with in-hospital mortality, including age, gender, polypharmacy, mental status, functional status, comorbidities, illness severity and presenting illness. However, measures of function and cognition of the elderly were those that were strongly related to in-hospital mortality [6]. Moreover, during the last years, several studies have included parameters such as components of comprehensive geriatric assessment, nutritional status, frailty and sarcopenia as factors related to mortality in elderly hospitalized patients [7-12].

We conducted this study in order to add to the bibliography findings regarding the relationship between in-hospital mortality and patients’ demographics and medical-functional status, as it is evaluated in the emergency department.

## Materials and methods

Sample, tools and data collection

A cross-sectional study was conducted in General and Oncological Hospital of Kifissia “Agioi Anargyroi” from September 2020 to December 2021, among older persons who were consecutively admitted through the emergency department.

On patients’ admission, a form was addressed to the patients’ demographic data (age, gender, marital status, educational level), comorbidities, number and type of drugs in use, body mass index (BMI), disease severity at admission, reason for hospitalization, frailty and cognitive status and dependency on activities of daily living. Information about patients was obtained by asking either the patients or their relatives when patients were not able to communicate.

Disease severity at admission was assessed using the quick Sequential Organ Function Assessment (qSOFA) score, which was introduced by the Sepsis-3 group in 2016 as an initial way to identify infected patients at high risk of mortality [13]. The scoring has also been used to assess disease severity in patients with heart failure and in adult patients, regardless of whether they had an infection or not [14,15].

Frailty was assessed using the Greek version of the revised 9-point Clinical Frailty Scale (CFS) [16,17-19]. The 7-point Global Deterioration Scale (GDS) was used for the evaluation of cognitive status, activities of daily living were evaluated by using Barthel Index (BI) and, for the measurement of comorbidity, the Charlson Comorbidity Index (CCI) was used [20-22]. CFS, BI, GDS and CCI were estimated for the premorbid patients’ status, prior to the onset of acute illness that led the patient to the hospital, based on the information received both from the patients and/or their relatives and from the patients’ medical history.

A first ethical approval for the study was obtained from Institutional Ethical and Scientific Committee of General and Oncology Hospital of Kifissia “Agioi Anargyroi” (approval number 1494). A second one was obtained from Committee on Bioethics and Deontology of School of Medicine, National and Kapodistrian University of Athens (approval number 284). An informed written consent was obtained from the patients. When a patient was not able to communicate, the written consent was obtained from his or her relative. In the first page of the form, a cover letter explained the purpose of the study. Moreover, in the first page it was clearly stated that in reports resulting from this study, confidentiality and anonymity would be assured.

Statistical analysis

All analyses were performed using IBM SPSS Statistics for Windows, Version 22.0 (IBM Corp., Armonk, NY). Categorical data are expressed as counts and percentages. Normality of all continuous variables was assessed using the Shapiro-Wilk test. The continuous variables patients’ age, BMI, CCI, BI, CFS score, GDS score, qSOFA score and medications’ number had a non-Gaussian distribution, and they are expressed as median and interquartile range (IQR).

Differences between discharged and deceased patients were evaluated using the chi-square test for qualitative variables and Mann-Whitney U test for continuous variables. A p-value <0.05 was considered statistically significant. Variables that differed statistically significant between discharged and deceased patients were included in a separate binary logistic regression analysis, to identify the most important ones. Regarding the logistic regression model, the most important factors affecting the outcome are presented as odds ratios (OR), including 95% confidence intervals (CIs). A receiver operating characteristic (ROC) curve was used to analyse the prognostic value of CFS and qSOFA scores.

**Figure 1:**
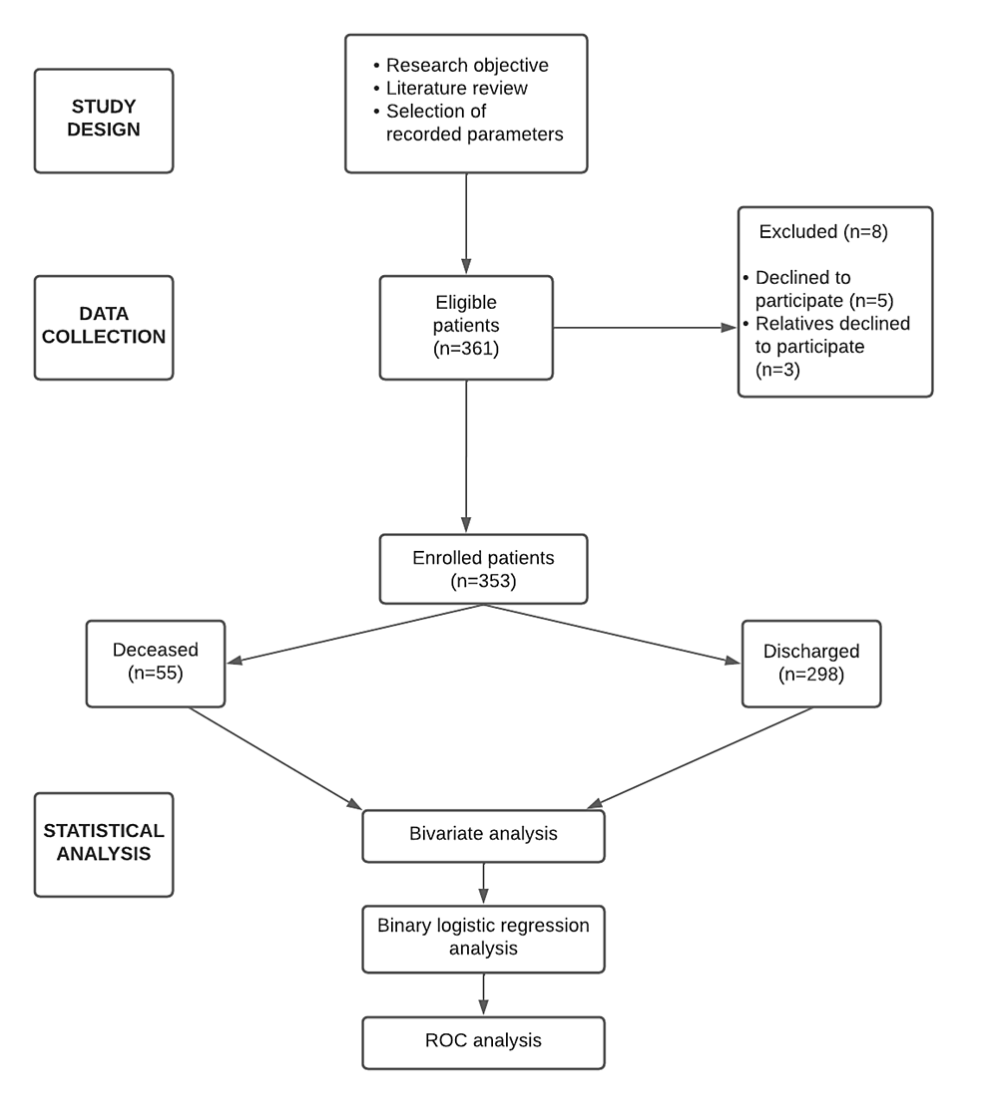
Methodology shown using a flowchart ROC: receiver operating characteristic

A flowchart showing the methodology is presented in Figure 1.

## Results

During the study period, 361 older patients were admitted to the medical unit via the emergency department. Five patients (three men and two women) denied to participate and for three more (one man and two women), who were unable to communicate, their relatives were reluctant to participate in the study. Finally, 353 patients enrolled in the study. The main reasons for being admitted to the hospital were anemia (72 patients, 20.4%), respiratory tract infection (60 patients, 17%), stroke (33 patients, 9.3%) and urinary tract infection (32 patients, 9.1%).

The median age of patients was 83 years (IQR 75-88). Among the participants, 169 were women (47.9%) and 184 men (52.1%). Patients’ characteristics are presented in Table 1.

**Table 1:** Patients’ characteristics IQR: interquartile range; CCI: Charlson Comorbidity Index; BMI: body mass index; BI: Barthel Index; GDS: Global Deterioration Scale; CFS: Clinical Frailty Scale; qSOFA: quick Sequential Organ Function Assessment; GFR: glomerular filtration rate

Characteristics (n=353)	
Gender	
Male	184 (52.1%)
Female	169 (47.9%)
Age (years), median (IQR)	83.00 (75.00-88.00)
Marital status	
Married	176 (49.9%)
Unmarried	9 (2.5%)
Divorced	12 (3.4%)
Widowed	156 (44.2%)
Educational level	
Primary	195 (55.3%)
Secondary	90 (25.5%)
Technological Education Institution	41 (11.6%)
University	27 (7.6%)
BMI, median (IQR)	22.30 (18.90-25.45)
BI, median (IQR)	85.00 (50.00-100.00)
CCI, median (IQR)	5.00 (4.00-7.00)
GDS score, median (IQR)	0.00 (0.00-2.00)
Medication number, median (IQR)	5.00 (4.00-7.00)
CFS score, median (IQR)	6.00 (3.00-7.00)
qSOFA score, median (IQR)	0.00 (0.00-1.00)
Aid use	
None	178 (50.4%)
Stick	69 (19.5%)
Frame	49 (13.9%)
Chairbound or bedridden	57 (16.1%)
Weight loss ≥5% in the last 6 months	
No	230 (65.2%)
Yes	123 (34.8%)
Presence of ulcer (pressure or vascular)	
No	317 (89.8%)
Yes	36 (10.2%)
Swallowing problems	
No	306 (86.7%)
Yes	47 (13.3%)
Active cancer	
No	275 (77.9%)
Yes	78 (22.1%)
Presence of any type of chronic respiratory disease	
No	273 (77.3%)
Yes	80 (22.7%)
Presence of any type of chronic heart disease	
No	179 (50.7%)
Yes	174 (49.3%)
Presence of any type of neurodegenerative disease or a history of stroke	
No	250 (70.8%)
Yes	103 (29.2%)
Presence of any type of chronic digestive disease	
No	296 (83.9%)
Yes	57 (16.1%)
Presence of chronic renal failure (GFR < 60)	
No	231 (65.4%)
Yes	122 (34.6%)

Differences between deceased and discharged patients are presented in Table 2.

**Table 2:** Comparison between deceased and discharged patients’ characteristics IQR: interquartile range; CCI: Charlson Comorbidity Index; BMI: body mass index; BI: Barthel Index; GDS: Global Deterioration Scale; CFS: Clinical Frailty Scale; qSOFA: quick Sequential Organ Function Assessment; NS: non-significant; GFR: glomerular filtration rate

	Deceased, n=55 (15.6%)	Discharged, n=298 (84.4%)	Statistical significance
Gender			NS
Males	30 (54.5%)	154 (51.7%)	
Females	25 (45.5%)	144 (48.3%)	
Age (years), median (IQR)	85 (76-89)	82 (75-87)	p=0.041 (U=6775.0)
Marital status			NS
Married	30 (54.5%)	146 (49.0%)	
Unmarried	2 (3.6%)	7 (2.3%)	
Divorced	0 (0.0%)	12 (4.0%)	
Widowed	23 (41.8%)	133 (44.6%)	
Educational level			NS
Primary	27 (49.2%)	168 (56.4%)	
Secondary	19 (34.5%)	71 (23.8%)	
Technological Education Institution	8 (14.5%)	33 (11.1%)	
University	1 (1.8%)	26 (8.7%)	
BMI	21.7 (18.3-26.7)	22.4 (19.1-25.4)	NS
BI, median (IQR)	40 (5-80)	90 (60-100)	p≤0.001 (U=4409.0)
CCI, median (IQR)	6 (5-8)	5 (4-7)	p=0.003 (U=6144.5)
GDS score, median (IQR)	2 (0-5)	0 (0-2)	p≤0.001 (U=5147.5)
Medication number, median (IQR)	6 (4-7)	5 (3-8)	NS
CFS score, median (IQR)	8 (6-9)	5 (3-7)	p≤0.001 (U=3443.5)
qSOFA score, median (IQR)	2 (1-2)	0 (0-1)	p≤0.001 (U=4094.5)
Aid use			p≤0.001 (χ^2^=33.873)
None	15 (27.3%)	163 (54.7%)	
Stick	9 (16.4%)	60 (20.1%)	
Frame	8 (14.5%)	41 (13.8%)	
Chairbound or bedridden	23 (41.8%)	34 (11.4%)	
Weight loss ≥5% in the last 6 months			NS
No	32 (58.2%)	198 (66.4%)	
Yes	23 (41.8%)	100 (33.6%)	
Presence of ulcer (pressure or vascular)			p≤0.001 (χ^2^=25.392)
No	39 (70.9%)	278 (93.3%)	
Yes	16 (29.1%)	20 (6.7%)	
Swallowing problems			p=0.001 (χ^2^=14.050)
No	39 (70.9%)	267 (89.6%)	
Yes	16 (29.1%)	31 (10.4%)	
Active cancer			NS
No	39 (70.9%)	236 (79.2%)	
Yes	16 (29.1%)	62 (20.8%)	
Presence of any type of chronic respiratory disease			p=0.042 (χ^2^=3.765)
No	37 (67.3%)	236 (79.2%)	
Yes	18 (32.7%)	62 (20.8%)	
Presence of any type of chronic heart disease			NS
No	26 (47.3%)	153 (51.3%)	
Yes	29 (52.7%)	145 (48.7%)	
Presence of any type of neurodegenerative disease or a history of stroke			p=0.004 (χ^2^=8.352)
No	30 (54.5%)	220 (73.8%)	
Yes	25 (45.5%)	78 (26.2%)	
Presence of any type of chronic digestive disease			NS
No	47 (85.5%)	249 (83.6%)	
Yes	8 (14.5%)	49 (16.4%)	
Presence of chronic renal failure (GFR < 60)			NS
No	35 (63.6%)	196 (65.8%)	
Yes	20 (36.4%)	102 (34.2%)	

Deceased patients were more probable to suffer from chronic respiratory (p=0.042, χ^2^=3.765) or chronic neurological disease (p=0.004, χ^2^=8.352), to report swallowing problems (p=0.001, χ^2^=14.050), to have pressure or vascular ulcers (p≤0.001, χ^2^=25.392) and to use walking aid (p≤0.001, χ^2^=33.873). Moreover, they were more probable to be older in age (p=0.041, U=6775.0), to have a higher qSOFA score at admission (p≤0.001, U=4094.5) and to have higher premorbid CFS (p≤0.001, U=3443.5), GDS (p≤0.001, U=5147.5), CCI (p=0.003, U=6144.5) and lower BI (p≤0.001, U=4409.0) scores.

A binary logistic regression was performed to ascertain the effects of the statistically significant variables on the likelihood of patients’ death. The logistic regression model was statistically significant, χ^2^(11) = 80.187, p≤0.001. The model explained 35.1% (Nagelkerke’s R^2^) of the variance in patients’ death and correctly classified 85.5% of cases. An increasing premorbid CFS score (p=0.001, OR=1.549, 95% CI 1.204-1.994) and a higher qSOFA score at admission (p=0.001, OR=1.895, 95% CI 1.282-2.802) were associated with an increased likelihood of patients’ death. In Table 3, the full model results are presented.

**Table 3:** Summary of binary logistic regression analysis B: regression coefficient; SE: standard error; Wald: Wald’s statistic; Sig.: p-value; Exp(B): odds ratio; CI: confidence interval; CCI: Charlson Comorbidity Index; GDS: Global Deterioration Scale; qSOFA: quick Sequential Organ Failure Assessment; BI: Barthel Index; CFS: Clinical Frailty Scale

	B	SE	Wald	Sig.	Exp(B)	95% CI for Exp(B)	
						Lower	Upper
Age	0.018	0.023	0.639	0,424	1.019	0.974	1.065
Walking aid	-0.238	0.268	0.786	0.375	0.789	0.466	1.333
CCI	0.018	0.085	0.046	0.830	1.019	0.862	1.204
GDS	0.179	0.125	2.048	0.152	1.196	0.936	1.527
qSOFA	0.639	0.199	10.275	0.001	1.895	1.282	2.802
BI	-0.004	0.011	0.103	0.749	0.996	0.975	1.019
Ulcers	0.724	0.486	2.226	0.136	2.064	0.797	5.345
Swallowing ability	-0.178	0.475	0.141	0.707	0.837	0.330	2.121
Respiratory disease	0.662	0.396	2.793	0.095	1.939	0.892	4.218
Neurological disease	-0.289	0.467	0.382	0.536	0.749	0.300	1.871
CFS	0.438	0.129	11.561	0.001	1.549	1.204	1.994

**Figure 2:**
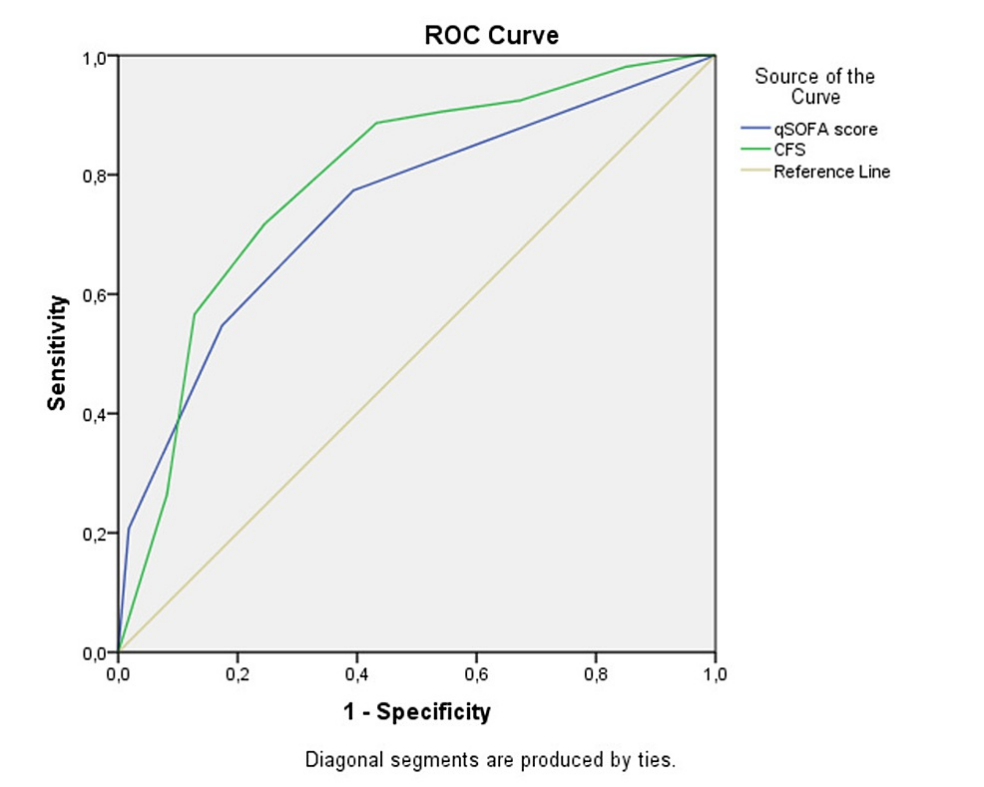
Area under the curve of the ROC curve analysis with respect to factors predicting mortality ROC: receiver operating characteristic; qSOFA: quick Sequential Organ Failure Assessment; CFS: Clinical Frailty Scale

When we used the ROC curve to analyse the prognostic value of qSOFA and CFS scores, we found that the classifiers had almost similar area under the curve (AUC) scores, with CFS performing slightly better. More specifically, our ROC analysis indicated that both CFS (AUC 0.79 [95% CI 0.73-0.85], p=0.001) and qSOFA (AUC 0.75 [95% CI 0.67-0.83], p=0.001) showed moderate accuracy for predicting inpatients’ mortality (Figure 2).

## Discussion

In this study, we evaluated numerous indices in order to identify factors that were associated with in-hospital mortality in elderly persons. The most significant were the premorbid patients’ functional status as assessed with the CFS and the disease severity at admission as assessed with the qSOFA score. This corroborates the statement that in elderly persons, two sources of risk are important: risk that arises from the illness or injury event, and risk that arises from a patient’s underlying health status before the acute event [3].

Regarding functional status, in a review of factors that affected the outcome in older patients admitted to the hospital, it was highlighted that there was a strong relationship between functional status and mortality [6]. Regarding CFS specifically, a previous scoping review revealed that it was highly predictive of mortality in multiple settings, including hospital [23].

For the evaluation of disease severity at admission, we used the qSOFA score. The qSOFA score has been originally developed for sepsis patients and it has been associated with mortality in old and very old patients with suspected infection [13,24]. However, it has also been used to assess disease severity in patients with heart failure and in adult admitted patients, both with and without suspected infection [14,15]. In both of these cases, increased qSOFA scores were associated with increased mortality in patients with heart failure and in admitted patients regardless of whether they had an infection or not. Previous studies that used other measures of illness severity to predict hospitalization outcomes in older persons showed a significant relationship of illness severity with mortality [25,26].

In general, in previous studies dealing with mortality prediction in elderly hospitalized patients, either the analysis laboratory variables were included or studies were conducted before the implementation of tools such as CFS for the assessment of frailty [27,28]. Or, they did not include disease severity at admission among the evaluated variables [7,8,10-12]. Hence, their results are not directly comparable with ours. However, Romero-Ortuno et al. in a study concluded that frailty and acute illness severity were independently associated with inpatient mortality, a result that is in line with ours [9].

Limitations

First, the study sample consisted of hospitalized patients, and hence, results concerning the prevalence of frailty and other study sample characteristics cannot be generalized for the whole community. Second, the cross-sectional design of the study does not allow to conclude causal relationships. Finally, although the study was conducted only in a tertiary care hospital and included only patients of one internal medicine department, we believe that patients’ profile was similar to that of patients attending the emergency department of other tertiary hospitals. Therefore, we consider that the sample is representative of this patient population.

## Conclusions

This study strengthens the perception of premorbid frailty and disease severity at admission as factors closely related to mortality in hospitalized elderly patients. Simple measures, such as CSF and qSOFA scores, may help in identifying in the emergency department elderly patients in need of particularly attention and care, in order to manage them appropriately and to provide them timely interventions. These tools are simple, and their use would be of great benefit to emergency physicians as the scores can be rapidly calculated for all emergency department elderly patients without the need for any laboratory or other tests.
